# Dr. George Crane

**Published:** 1916-03

**Authors:** 


**Dr. George Crane** (not listed in Polk nor State Directory), died at Addison, N. Y., Jan. 9, aged 76.

